# Synchronizing LLM-based semantic knowledge bases via secure federated fine-tuning in semantic communication

**DOI:** 10.3389/frai.2025.1690950

**Published:** 2025-10-24

**Authors:** Long Li, Yuanhang He, Rui Xu, Bei Chen, Boyu Han, Yuanyuan Zhao, Jianhua Li

**Affiliations:** 1 Shanghai Key Laboratory of Integrated Administration Technologies for Information Security, School of Computer Science, Shanghai Jiao Tong University, Shanghai, China; 2 National Key Laboratory of Security Communication, Chengdu, China

**Keywords:** semantic communication, large language model, semantic knowledge bases, homomorphic encryption, federated fine-tuning

## Abstract

Semantic communication (SemCom) has seen substantial growth in recent years, largely due to its potential to support future intelligent industries. This advancement hinges on the construction and synchronization of robust semantic knowledge bases (SKBs) across multiple endpoints, which can be achieved through large language models (LLMs). However, existing methods for constructing and synchronizing LLM-based SKBs often face numerous security threats, such as privacy leakage and poisoning attacks, particularly when federated fine-tuning is employed to update LLM knowledge bases. To address these challenges, we propose a novel Secure Federated Fine-Tuning (SecFFT) scheme for synchronizing LLM-based SKBs in semantic communication. First, we incorporate homomorphic encryption into SecFFT to ensure the secure synchronization of model parameters. Second, to enhance the trustworthiness of participants against poisoning attacks, we introduce a residual-based access control mechanism, where only participants with low residuals are authenticated to participate in updating the knowledge base. This mechanism is combined with a hash-based message authentication code. Third, we design a self-adaptive local updating strategy to minimize the impact of poisoned model parameters on benign participants, which is crucial for strengthening the robustness of LLM-based knowledge bases against poisoning attacks. Extensive experiments, conducted using four different datasets from the GLUE benchmark, demonstrate that SecFFT can securely synchronize distributed LLM-based SKBs while maintaining high accuracy (98.4% of the performance of the original federated LoRA), with an acceptable additional cost.

## 1 Introduction

**Figure 1:**
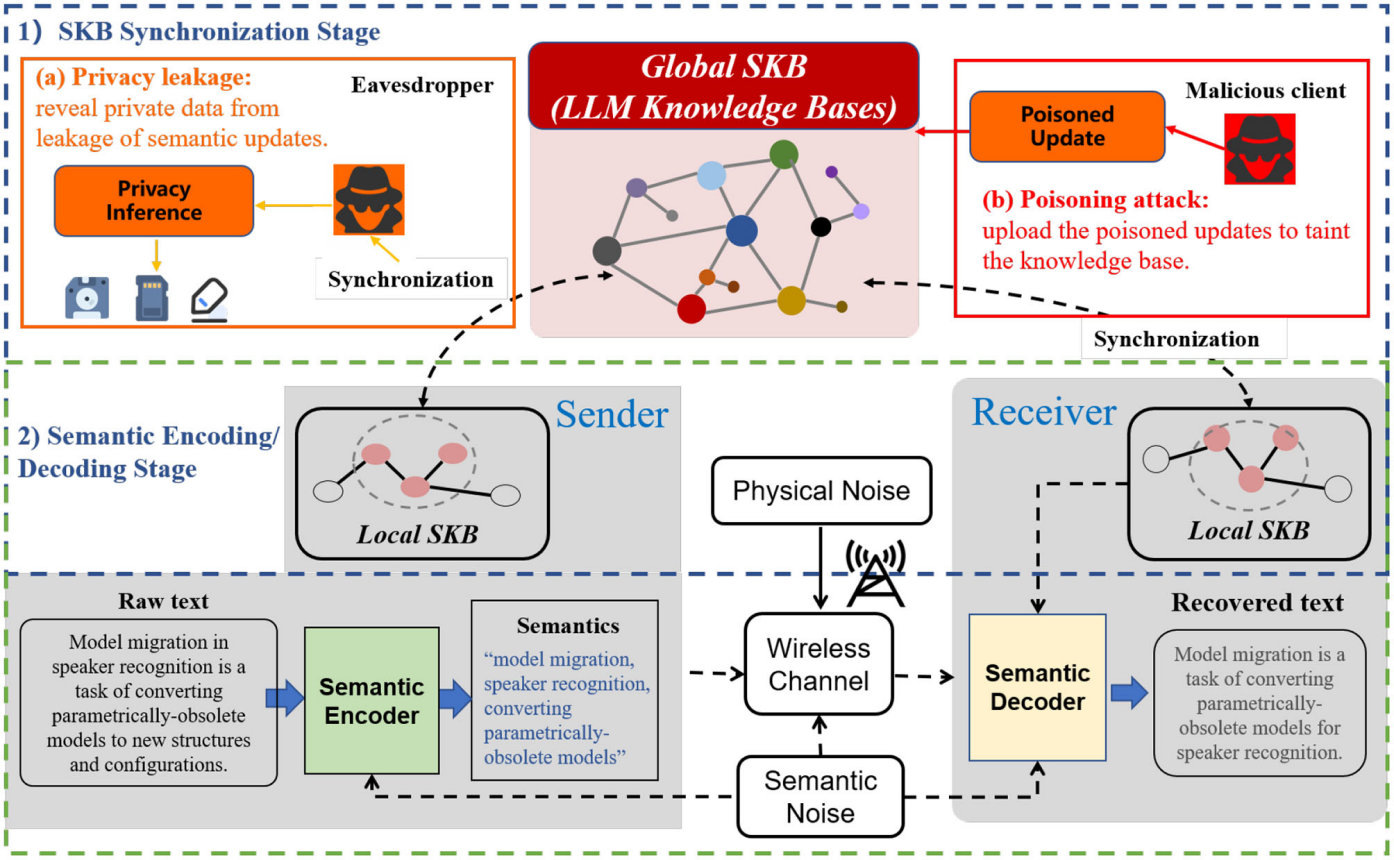
The universal framework of SemCom and its security threats. Therein, the knowledge base synchronization stage is exposed to privacy leakage and poisoning attacks.

Semantic communication (SemCom) is anticipated to become a pivotal paradigm in 6G networks, owing to its efficient information transmission, adaptability, and capacity to support complex application scenarios (Yang et al., 2024). At its core, SemCom focuses on extracting the “meaning” of the message sent from the source, and “translating” this semantic content at the destination based on a shared semantic knowledge base (SKB) between the sender and receiver, thereby reducing the volume of data transmitted. The universal framework of SemCom is depicted in Figure 1. In the SemCom architecture, the semantic encoder on the sender's side, guided by its local SKB, extracts semantics that convey background knowledge and context-relevant information from the raw text. Upon receiving the transmitted semantics through the wireless channel, the receiver employs a semantic decoder, also directed by its local SKB, to reconstruct the original text. SKB, serving as a representation of the knowledge space across multiple endpoints in SemCom, is a crucial solution for the generic extraction and recognition of semantic elements. Knowledge management in SemCom encompasses the creation, sharing, and updating of SKBs (Liang et al., 2024). Knowledge synchronization, primarily signifying the SKB updating procedure, is employed to align the SKBs of the semantic encoder and decoder, reducing the semantic gap between local SKBs and preventing miscommunication between nodes. This process is vital for strengthening semantic alignment between local SKBs and establishing a unified global SKB.

The large language model (LLM) has demonstrated remarkable compatibility with semantic communication (SemCom) systems for text transmission. Transformer-based LLMs are extensively utilized within the SemCom architecture (Guo et al., 2023; Jiang et al., 2023, 2024; Zhao et al., 2024), owing to their capacity to capture contextual relationships through the attention mechanism. Given that the parameter space of LLMs effectively captures conceptual relationships and factual knowledge, LLMs often serve as a semantic knowledge base (SKB). They are distributed to each client to extract semantics and reconstruct data (Lu et al., 2024). However, current methodologies fail to address security threats associated with the use of federated fine-tuning to synchronize LLM-based knowledge bases. Two primary threats emerge: (1) attacks during the SKB synchronization stage, and (2) attacks during the semantic encoding/decoding stage. In the latter stage, adversaries may execute adversarial attacks, model inversion attacks, and membership inference attacks on the LLM-driven semantic encoder/decoder. During the SKB synchronization phase, two primary threats are considered: privacy leakage and poisoning attacks. Privacy leakage refers to attacks where adversaries eavesdrop on the communication channel between the server and clients, gaining access to sensitive information such as updated model weights. Poisoning attacks involve adversaries surreptitiously modifying training samples or deliberately altering local model weights (Li et al., 2022).

In our approach, we integrate the LLM as a unified semantic processor, performing operations such as semantic encoding, decoding, and knowledge retrieval. Specifically, we leverage the LLM inference process for semantic encoding and decoding. To enhance the adaptability of the LLM to multi-round communication scenarios and optimize performance for specific semantic encoding/decoding tasks, LLM-based SKBs should undergo fine-tuning, rather than relying solely on the foundation model. However, the semantic gap between LLM-based SKBs can introduce ambiguity in the communication process. To mitigate this, we propose a secure federated fine-tuning framework designed to synchronize the knowledge space of distributed clients.

As depicted in Figure 1, we highlight the potential security threats associated with the synchronization of LLM-based SKBs in SemCom. Inquisitive adversaries can uncover local knowledge from leaked weight updates using the deep leakage gradient (DLG) (Zhu et al., 2019). Moreover, malicious actors can exploit system vulnerabilities to launch poisoning attacks, thereby disrupting the synchronization process and exacerbating the semantic gap between the local SKBs of different clients, ultimately leading to semantic errors within the SemCom framework. In this paper, we introduce a novel Secure Federated Fine-Tuning (SecFFT) scheme designed to synchronize LLM-based SKBs in SemCom. SecFFT enhances SemCom's resilience against attacks targeting the SKB synchronization phase. We summarize our key contributions as follows:

- We present the SecFFT scheme, a novel secure federated fine-tuning approach to synchronize LLM-based semantic knowledge bases in semantic communication systems. SecFFT incorporates three primary security mechanisms: (1) semantic-based homomorphic encryption, (2) residual-based access control, and (3) a self-adaptive updating strategy. Through SecFFT, both privacy leakage and poisoning attacks are mitigated with minimal additional cost.
- The semantic-based homomorphic encryption mechanism selectively encrypts high-level LLM parameters containing significant semantic content, leaving low-level parameters unencrypted. This enhances confidentiality during the knowledge base synchronization phase, focusing encryption on the parameters crucial for the model's semantic comprehension.
- In contrast to existing random participant selection methods in SKB synchronization, we introduce a residual-based access control mechanism with a hash-message-code-based authorization and authentication pipeline. This mechanism selectively authorizes nodes with low residuals to participate in the SKB synchronization process. Additionally, we propose a self-adaptive local updating strategy that prevents local models from being tainted by poisoned model parameters.
- We conduct extensive experiments to evaluate the performance of SecFFT, along with a comprehensive security analysis, demonstrating its superiority over existing methods.

The remainder of the paper is organized as follows. Section 2 reviews existing methods for LLM-based SKB synchronization, explores parameter-efficient federated fine-tuning approaches, and discusses the associated threats and defenses. Section 3 introduces the fundamental concepts and definitions necessary for constructing the SecFFT scheme. Section 4 provides an overview of SecFFT and details its three components designed to address the two primary threats encountered during the SKB synchronization phase. Section 5 presents the experimental setup and evaluation results. Finally, Section 7 concludes the paper.

## 2 Related work

### 2.1 Toward LLM-based semantic knowledge bases

With the widespread adoption and versatility of generative artificial intelligence (GAI), the field of semantic knowledge base (SKB) synchronization has increasingly focused on large language model (LLM)-enabled approaches. The authors in Guo et al. (2023) proposed a semantic importance-aware communication scheme based on pre-trained language models to enhance energy efficiency. In Jiang et al. (2023), a multimodal semantic communication framework was introduced, alongside the construction of a personalized SKB based on LLM, enabling users to create and maintain personalized semantic extraction and recovery, effectively addressing semantic ambiguity. In Jiang et al. (2024), an LLM-based semantic communication framework was developed with an attention-based semantic integration mechanism that automatically assigns weights to semantic segments. Additionally, an adaptive semantic compression encoding method was proposed to eliminate redundant information within semantic features, thereby reducing communication overhead. While most existing GAI-based methods demonstrate impressive performance, they fail to adequately address security and privacy concerns. In contrast, as demonstrated in Table 1, our approach successfully establishes a secure SKB synchronization scheme and safeguards against potential security and privacy threats.

**Table 1:** Summaries of differences between existing GAI-enabled knowledge base synchronization methods and ours.

**Properties**	**SIAC (Guo et al., 2023)**	**LAM-SMC (Jiang et al., 2023)**	**LAM-SC (Jiang et al., 2024)**	**SecFFT (ours)**
Base model	BERT	GPT-4	SAM	RoBERTa
Tuning method	×	Prompt tuning	×	FFA-LoRA
Knowledge update	×	×	×	✓
Privacy defenses	×	×	×	✓

× represents lack of the property while ✓ represents the opposite.

### 2.2 Parameter-efficient federated fine-tuning

Federated learning (FL) holds significant promise for the development of privacy-preserving large language models (LLMs), where distributed clients fine-tune or employ prompt engineering to train specific model parameters locally, and then aggregate them into a global LLM (Chen et al., 2023). To address challenges such as high communication costs between clients and servers, as well as the substantial computational load of local LLMs (Zhang et al., 2023), parameter-efficient fine-tuning (PEFT) was introduced. PEFT fine-tunes only a small set of lightweight parameters or a fraction of the total parameters for specific tasks, while most of the pre-trained model parameters remain frozen during the training process. This approach makes it feasible to apply FL to LLMs, reducing communication and computational burdens while maintaining the federated LLM's performance. Zhang et al. (2023) conducted experimental investigations into various PEFT methods, including adapter tuning (Houlsby et al., 2019), prefix tuning (Li and Liang, 2021), LoRA (Hu et al., 2021), and BitFit (Zaken et al., 2021) within an FL setting. They found that PEFT significantly reduced communication overhead and local storage costs, while still delivering acceptable federated LLM performance. Among these, LoRA stands out as the most efficient and promising PEFT method and has been further refined and adapted for federated LLM applications. The authors of Bai et al. (2024) introduced FlexLoRA, a LoRA-based method that adjusts ranks according to local client resources, enhancing the resulting model's generalization ability. Additionally, Babakniya et al. (2023) proposed SLoRA, a method that modifies the initialization of matrices to bridge the performance gap between PEFT and full fine-tuning. Our proposed scheme, SecFFT, builds upon FFA-LoRA (Sun et al., 2024), which fixes the initially nonzero matrix $A$ and only trains the initially zero matrix $B$, further reducing the number of trainable parameters.

### 2.3 Threats and defenses in federated large model

A federated large model is vulnerable to various security and privacy threats during both the training and inference stages, which correspond to the synchronization stage and the semantic encoding/decoding stage in LLM-enabled semantic communication, respectively. Our primary focus is on addressing the potential threats at the knowledge base synchronization stage, particularly privacy leakage and poisoning attacks. One significant form of privacy leakage is the Deep Leakage Gradient (DLG) attack (Zhu et al., 2019), which can reconstruct sensitive personal information from gradient updates (Khowaja et al., 2024) sent to the server. Additionally, a novel inference attack assumes the presence of a dishonest server, which can exploit flaws in the transformer architecture (Fowl et al., 2022) to extract private data from the client. Three widely researched defense mechanisms for mitigating privacy leakage include differential privacy (Wei et al., 2020), secure multi-party computation (Damgård et al., 2009), and homomorphic encryption (Rivest et al., 1978). Secure multi-party computation allows multiple parties to collaboratively compute a function without revealing private data, simulating the role of a trusted third party. Differential privacy (DP) (Wei et al., 2020) is typically implemented by applying random noise to gradient updates before they are uploaded to the server. While DP is effective in traditional FL settings, its performance degrades in federated LLMs due to the massive model parameters, as the noise added to the gradients rapidly becomes overwhelming. Homomorphic encryption (HE) enables computations to be performed on encrypted data, preserving privacy during the process. HE has been effectively applied in traditional FL settings as a defense mechanism for neural networks. For instance, the authors in Wang et al. (2023) proposed a privacy-preserving method using the Paillier algorithm, a classic homomorphic encryption scheme, within the FL process for a lightweight model, MobileNetV2. Similarly, Wibawa et al. (2022) employed the BFV scheme, another homomorphic encryption method, to safeguard federated training against privacy leakage. Given this context, we concentrate on homomorphic encryption and aim to enhance its applicability for federated LLMs, ensuring that it preserves the privacy of the LLM-driven knowledge base synchronization system.

Federated LLM is also exposed to poisoning attacks during the training stage. The authors in Li et al. (2024) proposed Fed-EBD, a new backdoor attack strategy for federated LLM, which implants a backdoor through a complex public dataset without eliminating the need for compromising any client or engaging long-term involvement in the training process. They further designed an attack (Li et al., 2023) without demanding the attacker to breach any client, which is proven to be effective in the realistic federated LLM environment. The authors in Wu et al. (2024) also proposed a novel backdoor attack for federated LLM. For research on defense methods against poisoning attacks in the scenario of federated LLM over the training stage, the authors in Zhou et al. (2024) proposed a pre-training strategy for foundation models through increasing the feature distance between samples and decreasing the feature distance between clean and poisoned samples without demanding clients to employ additional conduct. The authors in Huang et al. (2024) proposed a model-slicing-based secure distributed LLM framework, which employs lightweight encryption and a split fine-tuning scheme to secure the communication and mitigate the additional resource cost.

The aforementioned works attempt to conduct or defend against either privacy leakage or poisoning attacks. When it comes to the scenario of the combination of both threats, for example, when an attacker tries to reveal sensitive data from the poisoned model, relevant research is still lacking. Therefore, it is worthwhile to explore how to construct new defense mechanisms against the combination of both security threats during the training stage of federated LLM.

## 3 Preliminaries

SecFFT is built upon two core cryptographic primitives: the Paillier algorithm and the hash-based message authentication code (HMAC). On one hand, by leveraging the Paillier algorithm, a homomorphic encryption scheme, model weight parameters are encrypted during the synchronization process, allowing SecFFT to defend against privacy leakage threats during the SKB synchronization stage. On the other hand, HMAC, a widely adopted industry standard, is employed for message authentication and access control. The security of HMAC is reliant on the chosen hash function, with its security proof grounded in assumptions that are believed to hold in real-world scenarios.

### 3.1 Paillier algorithm

The Paillier algorithm (Paillier, 1999) is a partially homomorphic encryption scheme that provides additive homomorphism between the plaintext and ciphertext domains. Homomorphic encryption enables computations to be performed directly on encrypted data, with the result being equivalent to the corresponding operation on the plaintext. This homomorphic property makes the Paillier algorithm particularly valuable in cloud computing environments, where the protection of private data is essential. The Paillier algorithm comprises three key components: key generation, encryption, and decryption.

**Key generation**. Randomly select two large prime numbers *p* and *q*, which are of equal length and satisfy


$$\begin{array}{l} gcd\left(pq,\left(p-1\right)\left(q-1\right)\right)=1, \end{array}$$ (1)

where *gcd* refers to the common divisor of two numbers. We can Calculate *n* and λ as:


$$\begin{array}{l} n=pq, \end{array}$$ (2)

$$\begin{array}{l} \lambda =lcm\left(p-1,q-1\right), \end{array}$$ (3)

where *lcm* refers to the least common multiple. Then we randomly select $g\in {\mathbb{Z}}_{N^{2}}^{*}$. Let public key *pk* = (*n, g*), private key *sk* = (λ).

**Encryption**. Randomly select $r\in {\mathbb{Z}}_{n}^{*}$, and obviously $r\in {\mathbb{Z}}_{n^{2}}^{*}$. Calculate ciphertext *c* by:


$$\begin{array}{l} c=g^{m}r^{n}\;mod\;n^{2}, \end{array}$$ (4)

where *m* stands for the message to be encrypted.

**Decryption**. Let function $L\left(x\right)=\frac{x-1}{n}$. Calculate plaintext *m* by:


$$\begin{array}{l} m=\frac{L\left(c^{\lambda }\;mod\;n^{2}\right)}{L\left(g^{\lambda }\;mod\;n^{2}\right)}\;mod\;n. \end{array}$$ (5)

**Homomorphism property**. Paillier encryption satisfies the homomorphism property for addition:


$$\begin{array}{l} Enc\left(m_{1}\right)\times Enc\left(m_{2}\right)=Enc\left(m_{1}+m_{2}\right), \end{array}$$ (6)

where *Enc* refers to the encryption function and *m*_1_ and *m*_2_ refer to plain texts. The homomorphism property can be extended to scalar multiplication:


$$\begin{array}{l} Enc{\left(m_{1}\right)}^{k}=Enc\left(k\times m_{1}\right). \end{array}$$ (7)

The homomorphic property, which preserves operations, facilitates the aggregation of model weight updates on the server side in the form of ciphertexts.

### 3.2 Hash-based message authentication code

The Hash-based Message Authentication Code (HMAC) is a method for constructing a message authentication code using any cryptographically secure hash function, designed to verify message integrity and authenticate identity. In the HMAC framework, the sender computes the hash value of a combination of the arbitrary-length message and a pre-shared secret key shared among communication nodes, generating a fixed-length authentication code that is transmitted alongside the message. Upon receiving the message and the authentication code, the receiver performs the same operation with the shared secret key to verify the integrity and authenticity of the message. The calculation of HMAC involves two successive hash function operations:


$$\begin{array}{l} HV_{0}=H\left(\left(K⊕ipad\right)||M\right), \end{array}$$ (8)

$$\begin{array}{l} HV=H\left(HV_{0}||\left(K⊕opad\right)\right), \end{array}$$ (9)

where *H* denotes the hash function, *K* represents the randomly generated and pre-shared secret key, *M* is the message to be transmitted, ipad and opad are fixed constants, and ⊕ and || indicate the operations of XOR (exclusive OR) and concatenation, respectively.

## 4 Method

### 4.1 Overview and threat model

**Figure 2:**
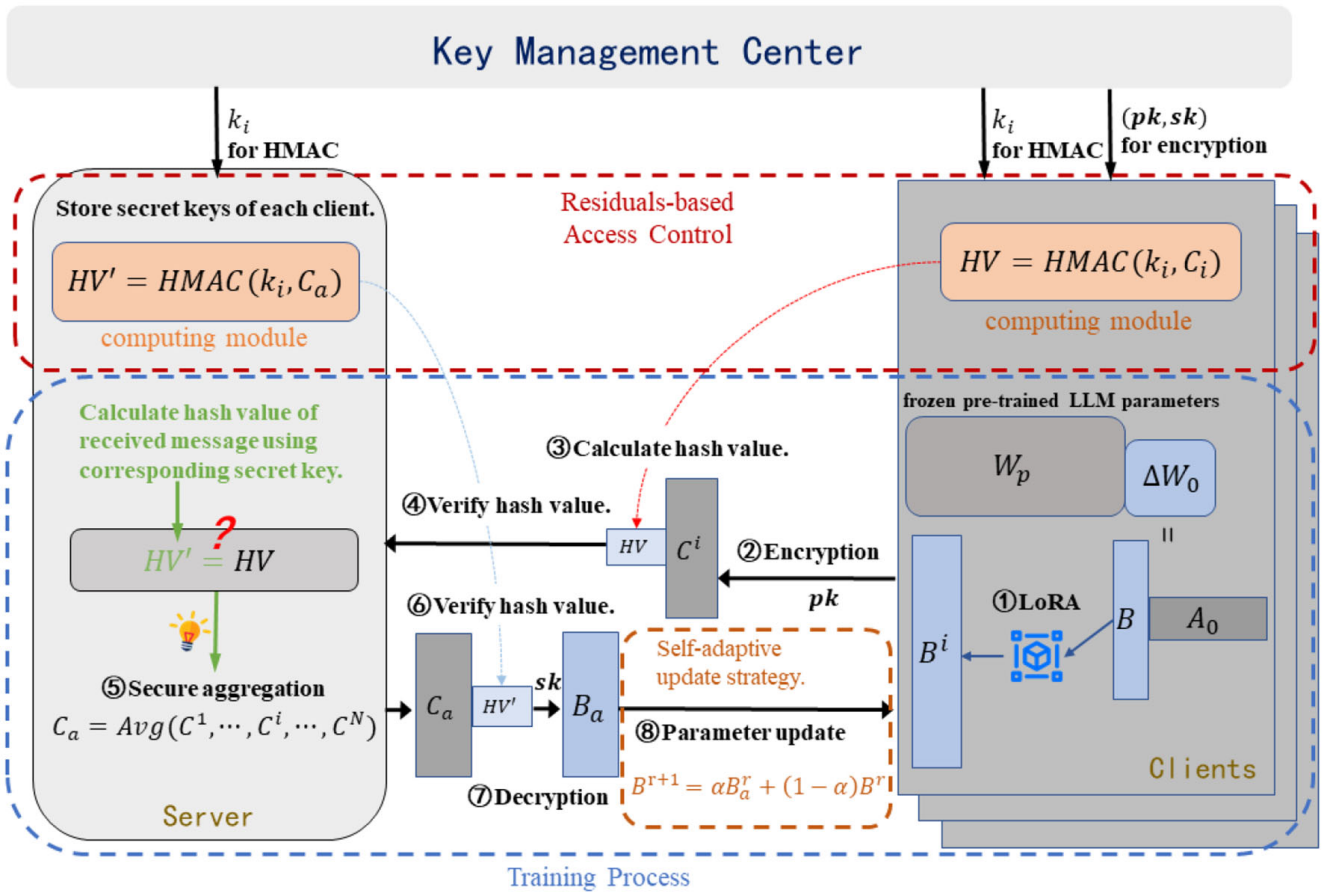
Overview of SecFFT and the communication diagram within the network. The privacy-preserving and robust property of the model is established by three components: semantic-based homomorphic encryption, residual-based access control mechanism, and self-adaptive updating strategy.

As depicted in Figure 2, we present an overview of SecFFT, where a large language model (LLM) serves as a distributed semantic knowledge base (SKB), and federated fine-tuning is employed as the synchronization method for the SKB across multiple clients.

Given the enormous number of parameters in LLMs, we utilize Low-Rank Adaptation (LoRA), the most efficient parameter-efficient fine-tuning method, to reduce both communication and computational costs while maintaining acceptable performance. The core idea of LoRA involves restricting the weight update in the model using a low-rank decomposition, *W*_*p*_+Δ*W* = *W*_*p*_+*BA*, where $W_{p}\in {\mathbb{R}}^{d\times k}$ represents the pre-trained weight matrix, which remains frozen during the training process. The update is constrained by the low-rank decomposition Δ*W* = *BA*, where *B*∈ℝ^*d*×*r*^ is initially set to zero, and *A*∈ℝ^*r*×*k*^ is initialized randomly using a Gaussian distribution. Since only the decomposed matrices are trained, the number of parameters requiring training is significantly reduced, particularly when *r*≪min(*d, k*). More specifically, the task-specific fine-tuning is performed over a much smaller set of parameters Θ where Θ≪Φ_0_, with Φ_0_ representing the pre-trained model weights. The task of finding ΔΦ involves optimizing over Θ (Hu et al., 2021):


$$\begin{array}{l} \underset{\Theta }{\max}\underset{\left(x,y\right)\in Z}{\sum }\overset{\left|y\right|}{\underset{t=1}{\sum }}\log\left(p_{\Phi_{0}+\Delta \Phi }\left(y_{t}|x,y_{<t}\right)\right), \end{array}$$ (10)

where ΔΦ = ΔΦ(Θ), $Z={\left\{\left(x_{i},y_{i}\right)\right\}}_{i=1,\ldots ,N}$ represents the training dataset of context-target pairs, and both *x*_*i*_ and *y*_*i*_ are sequences of tokens. Here, *p*_Φ_(*y*|*x*) = *p*_Φ_0_+ΔΦ_(*y*|*x*) is the pre-trained autoregressive language model parameterized by Φ.

The corresponding optimization objective for LoRA is to minimize the following loss function:


$$\begin{array}{l} L\left(\Theta ,A,B\right)=L_{task}\left(\Theta ,W+AB\right)+\lambda \left(||A|{|}_{F}^{2}+||B|{|}_{F}^{2}\right), \end{array}$$ (11)

where $L_{task}$ is the task-specific loss function, and λ represents the weight of the regularization term. $||\cdot |{|}_{F}^{2}$ denotes the Frobenius norm, used to regularize *A* and *B* in order to prevent overfitting.

Various studies are investigating more efficient and high-performance approaches to LoRA variants. Our encryption scheme is built upon FFA-LoRA (Sun et al., 2024). FFA-LoRA fixes the initially nonzero matrix *A*, training only the initially zero matrix *B*, thereby further reducing the number of trainable parameters. The formula for FFA-LoRA is *W*_*p*_+Δ*W* = *W*_*p*_+*BA*_0_, where *A*_0_ represents the frozen matrix during the training process. Accordingly, the optimization objective is to minimize the following loss function:


$$\begin{array}{l} L\left(\Theta ,A_{0},B\right)=L_{task}\left(\Theta ,W+A_{0}B\right)+\lambda ||B|{|}_{F}^{2}. \end{array}$$ (12)

We adopt FFA-LoRA as the synchronization method, integrating semantic-based homomorphic encryption, residual-based access control, and a self-adaptive local updating strategy as three defensive components to establish the privacy-preserving and robust properties of SecFFT.

**Threat model** We define the attacker's objectives and capabilities within the threat model. First, we consider an adversary A who can intercept the exchanged messages by eavesdropping on the communication channel during the SKB synchronization stage. The goal of A is to extract private data, originally contained in the distributed clients' datasets, from the intercepted messages. A is assumed to have complete access to all the communication content exchanged between the client and the server. Second, we consider an adversary B who manipulates a client to upload a malicious model weight, aiming to disrupt the knowledge base synchronization process. The objective of B is to degrade the performance of the global knowledge base, increase the semantic gap between clients' knowledge bases, and ultimately interfere with the communication process. Since B primarily conducts poisoning attacks, he does not have full control over the client, meaning he cannot access the secret keys stored within the client. Furthermore, we assume that the system model is secure against all potential attacks other than privacy leakage and poisoning attacks, with key distribution conducted before the synchronization process under perfectly secure conditions.

### 4.2 Semantic-based homomorphic encryption

As described in Section 4.1, we fine-tune only the initially zero matrix *B* to minimize the number of trainable parameters, thereby reducing computational costs. To safeguard against potential privacy leakage and enhance privacy preservation in SecFFT, we apply homomorphic encryption to the LoRA matrix *B*, specifically focusing on the high-level parameters in the last attention layer. While the model contains numerous parameters, we prioritize those that capture abstract semantic information, as they are critical to the model's overall task performance. In contrast, lower-level parameters mainly capture local features and detailed linguistic structures (Clark et al., 2019; Peters et al., 2018). Therefore, we apply homomorphic encryption only to the high-level parameters in the last attention layer, reducing computational costs while ensuring enhanced privacy protection. Specifically, for a large language model (LLM) with parameters Θ = {θ_1_, θ_2_, …, θ_*N*_}, the high-level parameters (*B*^*high*^) derived from the last attention layer's LoRA matrix are encrypted, ensuring privacy during the synchronization process.

Formally, as depicted in Algorithm 1, the key management center generates a pair of public and private keys (*pk, sk*) for a homomorphic encryption scheme and distributes the private key solely to the respective clients. This ensures the correctness of homomorphic aggregation, as clients must share the private key. After the local fine-tuning phase, the high-level weight update matrix $B_{i}^{high}$ of client *i* is homomorphically encrypted using the public key *pk*: $C_{i}^{high}=\text{Enc}\left(B_{i}^{high},pk\right)$. On the server side, upon successful message authentication, the encrypted weight parameters are permitted to enter the secure aggregation phase:


$$\begin{array}{l} C_{a}^{high}={\left(C_{1}^{\omega_{1}}○\cdots ○C_{i}^{\omega_{i}}\cdots ○C_{m}^{\omega_{m}}\right)}^{\frac{1}{\overset{m}{\underset{i=1}{\sum }}\omega_{i}}}, \end{array}$$ (13)

where ° and power represent the multiplication of corresponding elements of matrices, ω_*i*_ is the weight of client *i*, and *m* stands for the number of clients. For the remaining unencrypted low-layer weight parameters, we use FedAvg to aggregate:


$$\begin{array}{l} B_{a}^{low}=\frac{\overset{m}{\underset{i=1}{\sum }}\omega_{i}B_{i}}{\overset{m}{\underset{i=1}{\sum }}\omega_{i}}. \end{array}$$ (14)

After finishing secure aggregation, the server distributes the aggregated matrix to each client. The clients then decrypt the high-level averaged matrix using the private key, $B_{a}^{high}=\text{Dec}\left(C_{a}^{high},sk\right)$, and update all averaged parameters in their local knowledge base once the message has been authenticated. The semantic-based homomorphic encryption safeguards against privacy leakage within the synchronization system, as potential adversaries find it extremely difficult to extract meaningful information from the crucial high-level parameters in their encrypted form, thus ensuring the privacy-preserving integrity of SecFFT.

Algorithm 1 Semantic-based homomorphic encryption. 
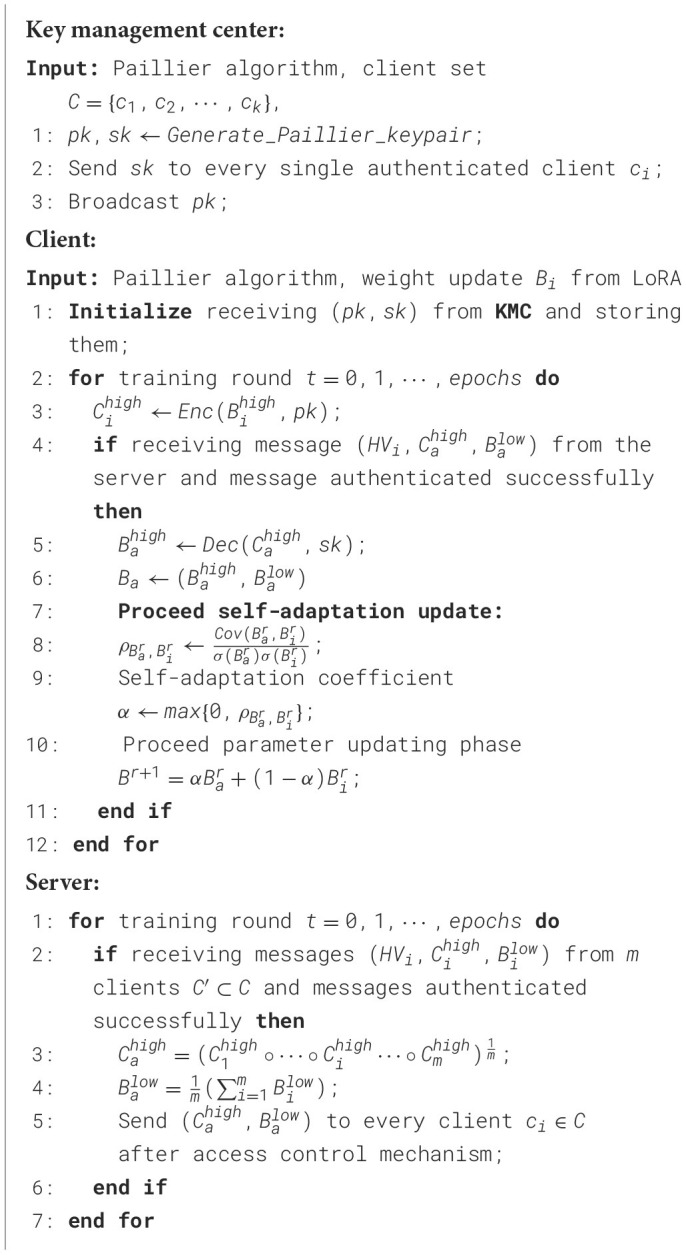

### 4.3 Residual-based access control

After receiving messages from clients, as illustrated in Algorithm 2, we implement HMAC-based access control, which is applied throughout the synchronization process to strengthen the privacy-preserving properties. Since HMAC requires a secret key, the key management center generates unique secret keys *k*_*i*_ for each client *i* and distributes them to the respective clients and the server prior to the synchronization process. The hash value (HV) of each message to be exchanged is computed using the HMAC algorithm and appended to the message. Furthermore, when a message, along with its HV, is received by a client or the server, the HV of the message is recalculated and compared with the attached HV. If the message is authenticated successfully, it proceeds to the residual-based access control process.

Algorithm 2 HMAC-based access control on client side. 
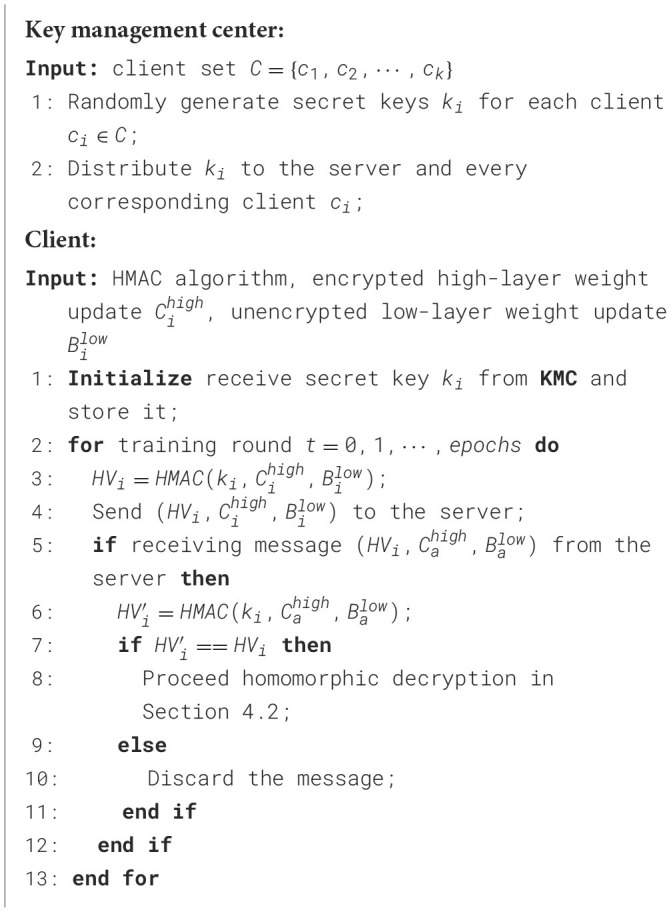

In the event that an authorized client is compromised and executes poisoning attacks on behalf of the adversary, we implement additional access control mechanisms based on residuals to enhance the robustness of SecFFT as depicted in Algorithm 3. Specifically, on the server side, we compute the median matrix *B*^*m*^ of the unencrypted low-layer matrices from all the authenticated model weights by:


$$\begin{array}{l} B^{m}=MED\left(B_{1}^{low},\cdots \;,B_{i}^{low},\cdots \;\right), \end{array}$$ (15)

where *MED* refers to the operation of computing the median value at each position across the matrices, and $B_{i}^{low}$ represents the unencrypted low-layer parameters from client *i*. Next, we calculate the residual matrix for each client as $B_{i}^{e}=B^{m}-B_{i}^{low}$ and compute its Frobenius norm $||B_{i}^{e}|{|}_{F}^{2}$. An ordered list of authenticated clients is then created based on the Frobenius norm of the residual matrices $||B_{i}^{e}|{|}_{F}^{2}$, and for the current communication round, the top *k* clients in the list are selected for the aggregation phase, where *k* is determined based on the server's computational resources.

Algorithm 3 Residuals-based access control on server side. 
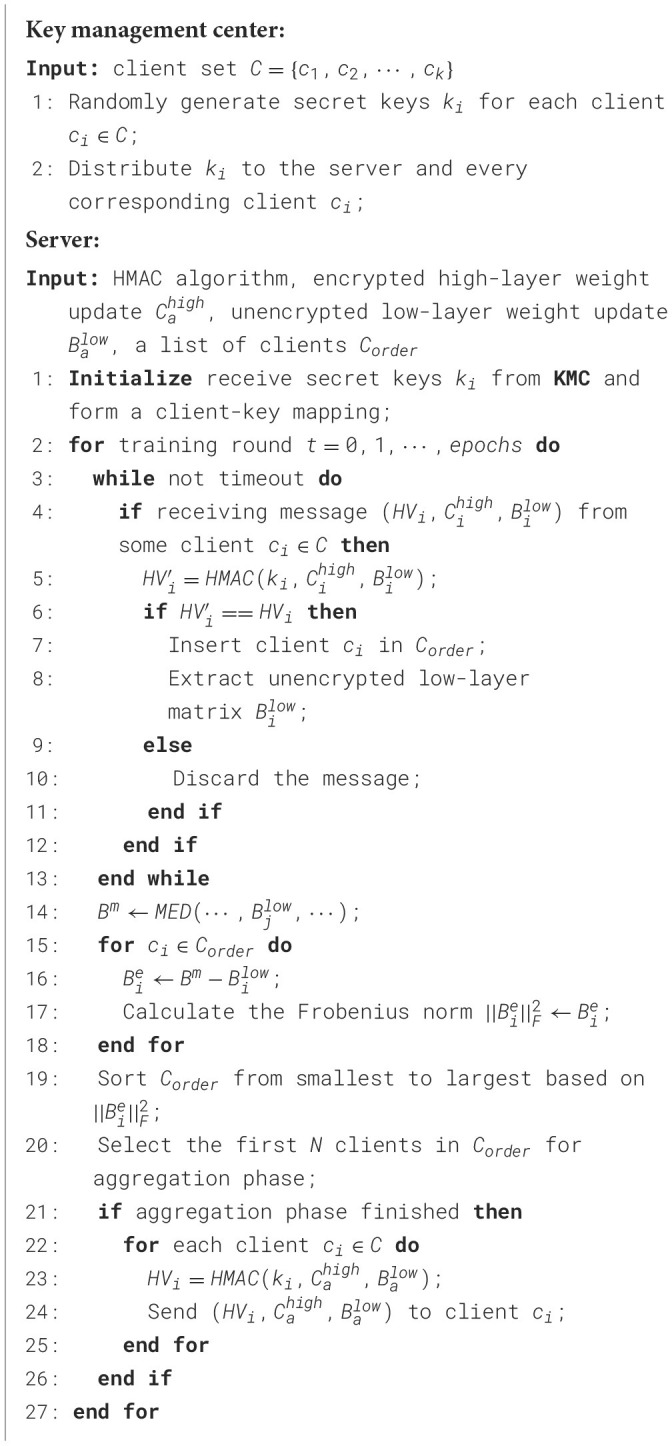

### 4.4 Self-adaptive updating strategy

In SecFFT, we implement a self-adaptive updating strategy as an integral part of the training process for the local SKB, further mitigating potential threats. Specifically, this approach is applied on the client side. Once the message from the server is authenticated and the weight parameters are decrypted, clients update the averaged matrix using the following formulas, rather than directly updating the local weight matrix:


$$\begin{array}{l} B^{r+1}=\alpha B_{a}^{r}+\left(1-\alpha \right)B^{r}, \end{array}$$ (16)

where *B*^*r*^ represents the resulting weight matrix from the *r*-th local training round, *B*^*r*+1^ denotes the initial weight matrix for the *r*+1-th local training round, and $B_{a}^{r}$ represents the global averaged weight matrix for the *r*-th training round. Additionally, $\alpha =\max\left\{0,\rho_{B_{a}^{i},B^{i}}\right\}$, where


$$\begin{array}{l} \rho_{B_{a}^{r},B^{r}}=\frac{Cov\left(B_{a}^{r},B^{r}\right)}{\sigma \left(B_{a}^{r}\right)\sigma \left(B^{r}\right)} \end{array}$$ (17)

represents the Pearson correlation coefficient between *B*^*r*^ and $B_{a}^{r}$. This strategy aims to adapt the weight matrix update process in the local SKB based on the relevance between *B*^*r*^ and $B_{a}^{r}$. In other words, the higher the correlation between the two matrices, the greater the contribution of the aggregated matrix to the updated matrix. Conversely, when the relevance is low, the resulting weight matrix incorporates fewer components of $B_{a}^{r}$ and more components of *B*^*r*^, or even entirely consists of *B*^*r*^.

The self-adaptive updating strategy helps mitigate poisoning attacks and strengthens the robustness of SecFFT from the perspective of the local client. For instance, if a client is compromised by an adversary, the attacker may conduct poisoning attacks or other malicious activities by uploading a poisoned model weight matrix to the server (since the homomorphic encryption key is public in the communication channel), thereby contaminating the final aggregated model weight. The poisoned matrix is expected to exhibit minimal relevance to the locally trained weight matrix, and we can neutralize the malicious impact of such poisoning attacks by reducing the contribution of the averaged matrix.

### 4.5 Security analysis

We provide a comprehensive security analysis of SecFFT from five key aspects:

**Preserving local data privacy:** The knowledge synchronization method ensures that distributed local data remains private throughout the synchronization process, preventing raw data from being exposed in a malicious network environment.

**Chosen-plaintext attack (CPA) security of paillier:** Clients within the network are unable to access parameters updated by other clients due to the HMAC-based access control mechanism, while the server can only receive the parameters in ciphertext form and is unable to decrypt them. Furthermore, the Paillier encryption algorithm satisfies CPA security (Li and Micciancio, 2021; Damgård et al., 2010), which is based on the semantic security of public-key cryptography and the assumption that clients and servers do not collude. This ensures that even if an attacker intercepts the encrypted parameters, they cannot derive the corresponding plaintext. Additionally, the security of HMAC relies on the security of the employed hash function and the strength of the shared secret key (Beringer et al., 2015). Thus, we ensure the security of the HMAC-based component in SecFFT by utilizing a secure hash function and generating sufficiently long secret keys.

**Noise-free encryption:** Homomorphic encryption does not introduce noise during the encryption/decryption process, ensuring that there is no loss in learning accuracy (Zhang et al., 2020).

**Semantic-aware encryption:** While we encrypt only a portion of the parameters due to computational constraints, we believe that the privacy-preserving property is still upheld. The parameters chosen for encryption, specifically those in the last attention layer, focus on semantic information and are more crucial for the semantic knowledge base, given their role in feature extraction and recovery within semantic communication.

**Robustness property:** The HMAC-based access control mechanism is applied throughout the synchronization process, enhancing the robustness of SecFFT. Moreover, SecFFT aligns with the assumption that the system is vulnerable, thereby challenging users and devices (Bandara et al., 2022). This implies that even if a potential adversary breaches the network, SecFFT prevents them from gaining default access privileges to other devices or applications within the network (Samaniego and Deters, 2018). By utilizing the residual-based access control mechanism and self-adaptive updating strategy, SecFFT limits the influence of any authenticated client on other clients, even if compromised.

In summary, SecFFT establishes a privacy-preserving and robust architecture.

## 5 Experiments

In this section, we present extensive experiments to evaluate SecFFT, which include a performance comparison (Section 5.2), a comprehensive ablation study (Section 5.3), and additional discussion (Section 6). All experiments are conducted using RoBerta-base (Liu et al., 2019), a widely used and robust model known for its versatility, as demonstrated in Pan et al. (2024) and Sun et al. (2020).

### 5.1 Experimental setup

#### 5.1.1 Dataset and non-IID partition

Our experiments utilize four datasets–RTE, MRPC, SST-2, and QNLI–taken from the GLUE benchmark (Wang et al., 2018), a standard framework for evaluating natural language tasks due to its diversity and complexity. The data distribution and evaluation metrics for these datasets are detailed in Table 2. For the non-IID partition, we follow the setup described in Lin et al. (2021), using the Dirichlet distribution to determine class priors for partitioning the datasets. Specifically, we sample the datasets by D~D(α), and distribute the partitioned dataset $D_{k}$ to the *k*-th client, where α represents the degree of non-IIDness.

**Table 2:** Dataset descriptions and statistics.

**Datasets**	**# Train (*N*)**	**# Dev. (*N*)**	**# Test (*N*)**	**Metrics**
RTE	2,241	249	277	Accuracy
MRPC	3,301	367	408	F1 score
SST-2	66,675	674	872	Accuracy
QNLI	103,695	1,048	5,463	Accuracy

#### 5.1.2 Implementation details

We integrate LoRA adapters into every attention layer of the RoBerta-base model to ensure its satisfactory performance. Given the substantial number of parameters that need to be updated, we apply homomorphic encryption only to the LoRA parameters in the final attention layer. FedAvg (McMahan et al., 2016) is used as the aggregation method for the unencrypted parameters, performing average addition during the aggregation process. For encrypted parameters, we leverage the homomorphism property and use modular multiplication as described in Equation (13) to align with FedAvg in the ciphertext domain. Furthermore, the implementation of LoRA and FFA-LoRA is based on *OpenDelta* (Hu et al., 2023), a plug-and-play framework designed for parameter-efficient fine-tuning.

### 5.2 Performance comparison

We compare the performance of SecFFT with original FedLoRA and FFA-LoRA on RTE, MRPC, SST-2, and QNLI. To ensure a fair comparison, we maintain the following settings: a local batch size of *B* = 32, a learning rate of η = 2*e*−5, 10 total communication rounds, 100 clients (which can serve as both transmitters and receivers in the semantic communication framework), and a LoRA adapter decomposition rank of *r* = 8. To achieve optimal performance for each dataset, we empirically set the local training epochs to 80 for RTE, 30 for MRPC, 60 for SST-2, and 25 for QNLI. Additionally, since data heterogeneity is a critical factor in federated fine-tuning, we also assess the performance of SecFFT across different non-IID partitioned datasets in this section, choosing the non-IID degree α from {0.1, 1.0, 10.0}. Regarding homomorphic encryption, since most parameters in the LoRA-B matrix have relatively small magnitudes and the Paillier algorithm is designed for integers, we scale the parameters up by multiplying by 1,000 before encryption and scale them down after decryption.

Table 3 presents a performance comparison between SecFFT, FedLoRA, and FFA-LoRA across four datasets with varying non-IID degrees. From the table, we observe that while the performance of SecFFT slightly lags behind the original FedLoRA, the performance gap remains acceptable. For instance, in QNLI, SecFFT's performance decreases by only 2.5% compared to FedLoRA, and by 0.3% compared to FFA-LoRA. Additionally, across all four tasks, SecFFT achieves 98.4% of the performance of FedLoRA. Notably, in RTE, SecFFT outperforms FedLoRA slightly. This discrepancy could be attributed to the fact that homomorphic encryption may alter the lower digits after the decimal point in the parameters, even after performing the scaling operation, which affects model accuracy. Additionally, all tuning methods tend to be more unstable on smaller datasets (Zhang et al., 2023; Chen et al., 2022). Excluding the RTE results, SecFFT still maintains 97.0% of the performance of FedLoRA and 99.9% of the performance of FFA-LoRA. It is important to highlight that SecFFT achieves competitive performance compared to FFA-LoRA, outperforming it on two out of four datasets. The above analysis is based on results obtained when α = 1.0.

**Table 3:** Comparisons of the state-of-the-art methods for bit accuracy w.r.t various distortion types.

**Dataset**	**RTE**			**SST-2**			**MRPC**			**QNLI**			**Avg**.	***Rel*.**
α	**0.1**	**1.0**	**10.0**	**0.1**	**1.0**	**10.0**	**0.1**	**1.0**	**10.0**	**0.1**	**/**	**/**	**/**	**/**
FedLoRA	47.3	60.6	57.0	50.9	92.9	92.7	81.2	85.8	88.4	83.8	88.7	90.0	**76.6**	100%
FFA-LoRA	47.3	50.5	56.7	66.9	91.7	91.7	81.2	81.2	81.5	74.4	86.7	87.7	**74.8**	97.7%
**SecFFT**	47.3	61.0	50.9	66.8	91.6	91.7	81.2	81.4	81.7	77.4	86.5	87.7	**75.4**	98.4%

We show the results on 32-bit watermarking messages. The bold results in this table are the average of the above four datasets. *Rel*. denotes the percentage of FFA-LoRA and SecFFT in terms of performance relative to FedLoRA.

Regarding the impact of data heterogeneity, a smaller α corresponds to a sharper non-IID distribution among clients. We observe that greater data heterogeneity leads to a decrease in SecFFT's performance. Specifically, when α decreases from 1.0 to 0.1 on relatively large datasets like SST-2 and QNLI, performance significantly deteriorates. This suggests that PEFT methods are more vulnerable to data heterogeneity and that handling complex data heterogeneity with fewer trainable parameters is challenging. Conversely, the performance results for α = 1.0 and α = 10.0 show minimal difference, indicating that the data heterogeneity between these two parameter settings does not have a significant impact. The effect of data heterogeneity on SecFFT is also illustrated in Table 3.

**Figure 3:**
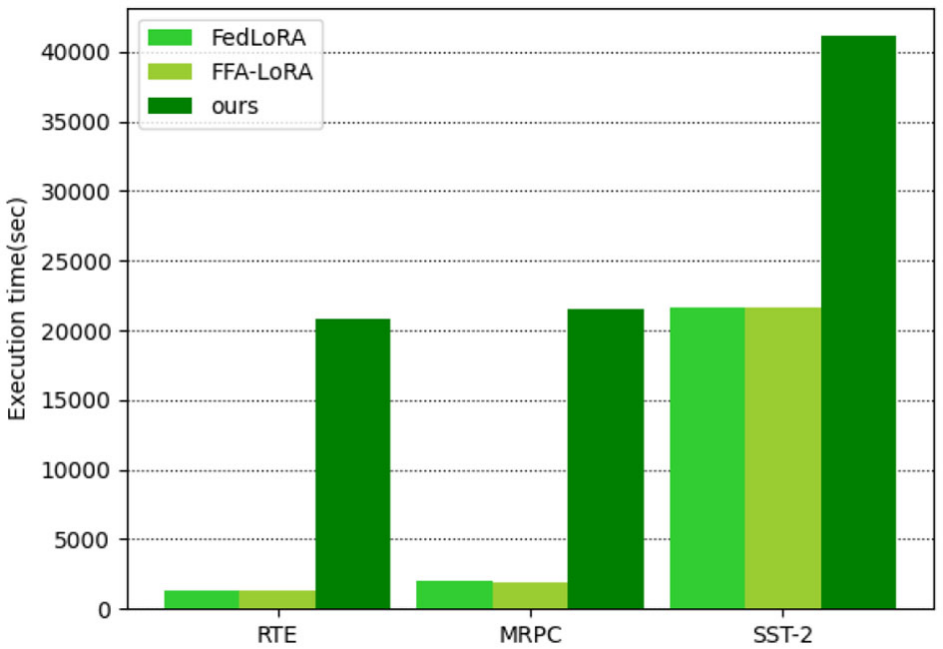
Execution time in seconds of SecFFT compared with federated LoRA and FFA-LoRA.

Furthermore, we evaluate additional costs induced by the privacy-preserving measurements in SecFFT from several aspects:

- Execution time. Homomorphic encryption, which plays a critical role in defending against privacy leakage risks in SecFFT, requires extensive modulo power operations that consume considerable time due to their inherent complexity. As a result, the overall execution time of SecFFT for 10 training rounds is significantly higher compared to FFA-LoRA and federated LoRA, as illustrated in Figure 3. However, we consider the increased execution time acceptable for two reasons. First, in semantic communication, SecFFT, which synchronizes the knowledge base across multiple endpoints in the network, is demand-driven and does not occur as frequently as the semantic encoding/decoding process. Thus, the impact of SecFFT on execution time is manageable within the broader context of the semantic communication procedure. Second, the performance and privacy security benefits provided by SecFFT, even at the cost of additional execution time, enhance the capabilities and efficiencies of the semantic encoder/decoder, improving the overall communication process. Therefore, we regard the additional execution time cost in SecFFT as acceptable within the entire semantic communication framework.
- Memory overhead. Since the local training process, encryption process, and decryption process are executed serially and locally across all the clients, the demand for memory resources of local clients hardly grows from FFA-LoRA with no privacy-preserving measurements. Besides, when it comes to larger network environments, the increase in the number of clients does not directly affect the local memory overhead for every single client. Therefore, the privacy-preserving components in SecFFT cause few increment of memory overhead.
- Synchronization cost. Since homomorphic encryption lengthens the ciphertext in bits compared to the plaintext, the number of data bytes to be exchanged is increased. Therefore, SecFFT has an additional synchronization cost than FFA-LoRA.

In summary, the results show that SecFFT maintains satisfactory performance (98.4% of the original FedLoRA) while introducing only acceptable additional costs. At the same time, it enhances privacy-preserving and robust properties to defend against various security threats. SecFFT effectively strikes a balanced trade-off between performance, additional costs, and privacy guarantees in practical applications.

### 5.3 Ablation study

#### 5.3.1 Impact of LoRA rank

The influence of LoRA rank on performance, excluding privacy measures, has been examined, with the conclusion that increasing rank does not necessarily enhance the information extracted from gradients (Sun et al., 2024). Further investigation is required to determine whether this conclusion remains valid when privacy measures are applied. Consequently, we assess the impact of LoRA rank on both performance and synchronization cost in SecFFT, selecting decomposition ranks from {2, 4, 8} while keeping all other parameters consistent across datasets.

**Figure 4:**
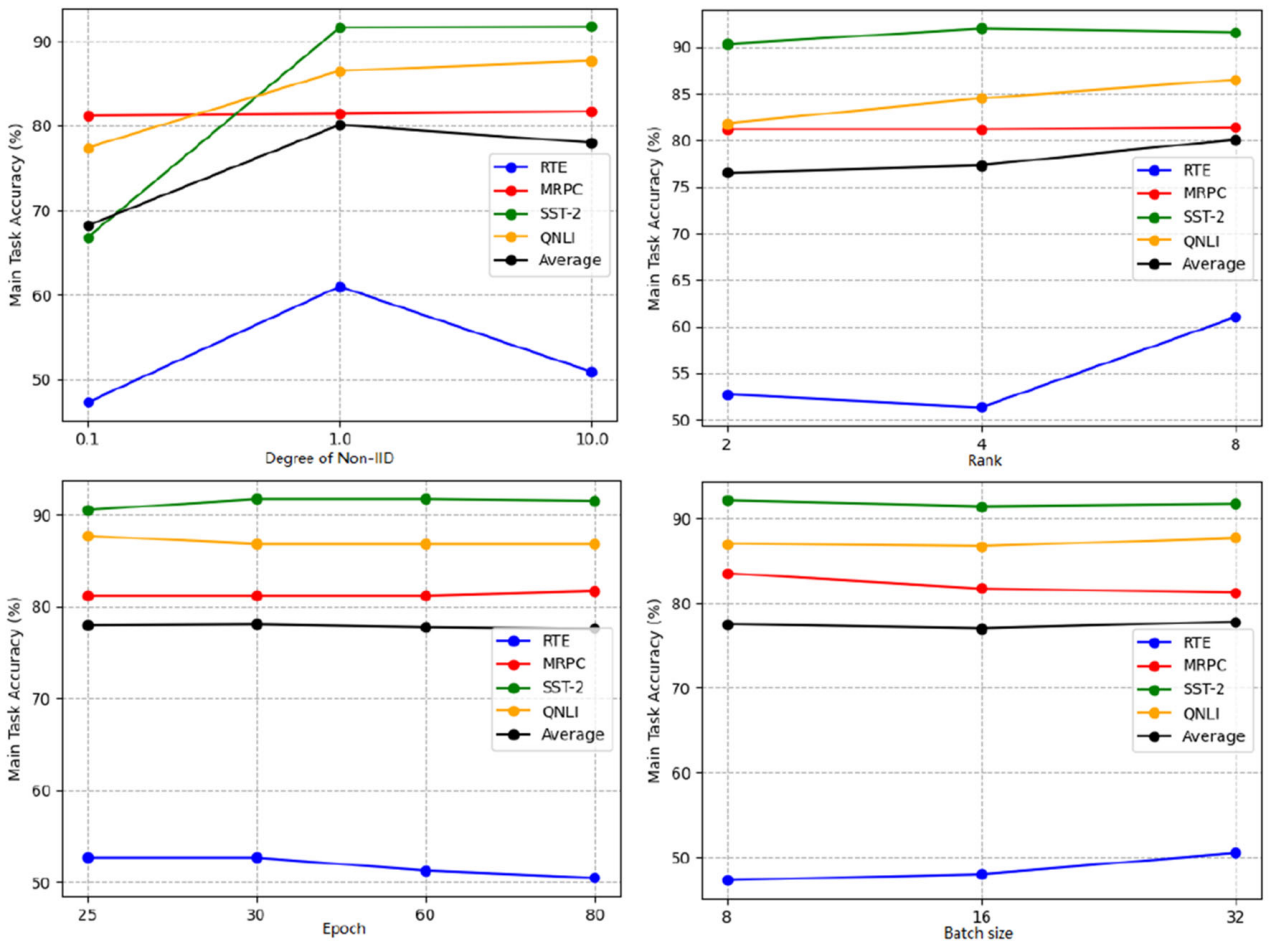
Main task accuracy (%) of SecFFT with different settings including degree of non-IID, LoRA rank, local training epoch, and batch size under different datasets.

The performance results are presented in Table 4 and Figure 4. It is evident that increasing the LoRA rank has a minimal impact on the performance of SecFFT with privacy-preserving measures across all datasets. Specifically, increasing the LoRA rank, which corresponds to a higher number of trainable parameters, does not necessarily improve performance. Therefore, the conclusion that LoRA rank has a negligible effect on the information extracted from gradients holds true in SecFFT with privacy measures.

**Table 4:** Main task accuracy (%) with different LoRA rank.

**Rank**	**RTE**	**MRPC**	**SST-2**	**QNLI**	**Avg**.
2	52.7	81.2	90.3	81.8	76.5
4	51.3	81.2	**92.0**	84.5	77.3
8	**61.0**	**81.4**	91.6	**86.5**	**80.1**

The bold values are the best results among different LoRA rank over four datasets and their average.

**Figure 5:**
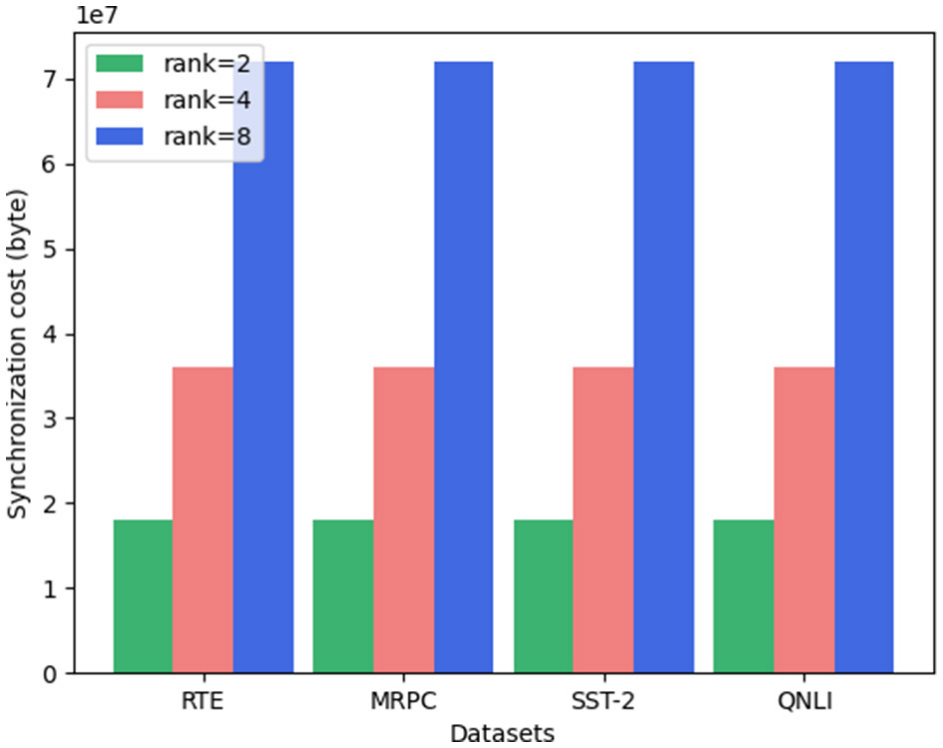
Synchronization cost of SecFFT with different LoRA rank under different datasets.

Additionally, we examine the synchronization cost for different LoRA ranks. As illustrated in Figure 5, the synchronization costs follow the order: “rank = 2” < “rank = 4” < “rank = 8” across all datasets. Notably, the synchronization cost exhibits a near-linear relationship with the LoRA rank. This is logical, as only the unfrozen parameters are exchanged, and the number of unfrozen parameters in our experimental setup is directly proportional to the LoRA rank. Hence, it is clear that increasing the LoRA rank leads to a corresponding rise in synchronization cost. In summary, while LoRA rank has little effect on the performance of SecFFT with privacy-preserving measures, it significantly influences synchronization cost.

#### 5.3.2 Impact of training epoch and batch size

We also investigate the influence of local training epochs and batch size on the performance of SecFFT without encryption. Specifically, we explore the impact of training epochs by selecting values from {25, 30, 60, 80}, while maintaining a batch size of 32 across all four datasets. For batch size, we consider values from {8, 16, 32}, setting the local training epoch to 80 for RTE, 30 for MRPC, 60 for SST-2, and 25 for QNLI.

The performance results of SecFFT with varying training epochs are presented in Table 5 and Figure 4. We observe that the optimal training epoch for each of the four datasets differs, largely aligning with the epoch settings in Section 5.2. However, this trend does not hold for RTE and MRPC. We hypothesize that this discrepancy is due to the relatively small size of the RTE and MRPC datasets, where increasing the number of training epochs can lead to overfitting, thus degrading performance.

**Table 5:** Main task accuracy (%) with different training epoch.

**Epoch**	**RTE**	**MRPC**	**SST-2**	**QNLI**	**Avg**.
25	**52.7**	81.2	90.5	**87.7**	78.0
30	**52.7**	81.2	**91.7**	86.8	**78.1**
60	51.3	81.2	**91.7**	86.8	77.8
80	50.5	**81.7**	91.5	86.8	77.6

The bold values are the best results among different training epoch over four datasets and their average.

The performance results of SecFFT with different batch sizes are shown in Table 6 and Figure 5. It is evident that, for most datasets, a larger batch size improves SecFFT's model performance. However, only two out of the four datasets fully conform to this observation. For SST-2, the performance difference between a batch size of 8 and 32 is negligible enough to be considered acceptable. For MRPC, we infer that this inconsistency may be due to the inherent instability of tuning methods on smaller datasets, as highlighted in previous studies (Zhang et al., 2023; Chen et al., 2022).

**Table 6:** Main task accuracy (%) with different batch size.

**Rank**	**RTE**	**MRPC**	**SST-2**	**QNLI**	**Avg**.
8	47.3	**83.5**	**92.1**	87.0	77.5
16	48.0	81.7	91.4	86.7	77.0
32	**50.5**	81.2	91.7	**87.7**	**77.8**

The bold values are the best results among different batch size over four datasets and their average.

## 6 Discussion

The versatility and efficiency of federated large models have led to their adoption in various scenarios beyond semantic communication, such as context information sharing. Since federated large model-based methods in these applications are also employed for the extraction, recovery, and exchange of semantic data, we believe that SecFFT can be seamlessly integrated into these methods without significant degradation in performance. Furthermore, SecFFT incorporates privacy-enhancing measures, strengthening its ability to defend against potential adversaries and attacks, thereby ensuring enhanced security in these settings.

## 7 Conclusion

This paper introduces SecFFT, a privacy-preserving and robust knowledge base synchronization scheme for LLM-enabled Semantic Communication (SemCom), designed to address potential security threats during the knowledge base synchronization stage. We leverage federated LLMs as the distributed knowledge base in SemCom and employ federated fine-tuning as the synchronization method. To establish a privacy-preserving architecture, we incorporate semantic-based homomorphic encryption into SecFFT to secure communication between the server and clients. Additionally, we propose a residual-based access control mechanism and a self-adaptive local updating strategy to further enhance the robustness of SecFFT. Extensive experiments were conducted to evaluate SecFFT's performance, with numerical results demonstrating that SecFFT maintains acceptable performance compared to methods lacking privacy-preserving measures, while achieving semantic security. Although the additional cost is acceptable, further improvements in efficiency remain a focus for future research.

## Data Availability

The original contributions presented in the study are included in the article/supplementary material, further inquiries can be directed to the corresponding author.

## References

[B1] Babakniya S. Elkordy A. R. Ezzeldin Y. H. Liu Q. Song K.-B. El-Khamy M. . (2023). Slora: federated parameter efficient fine-tuning of language models. arXiv [preprint]. arXiv:2308.06522. doi: 10.48550/arXiv.2308.06522

[B2] Bai J. Chen D. Qian B. Yao L. Li Y. (2024). Federated fine-tuning of large language models under heterogeneous language tasks and client resources. arXiv [preprint]. arXiv:2402.11505. doi: 10.48550/arXiv.2402.11505

[B3] Bandara E. Liang X. Shetty S. Mukkamala R. Rahman A. Keong N. W. . (2022). “Skunk—a blockchain and zero trust security enabled federated learning platform for 5g/6g network slicing,” in 2022 19th Annual IEEE International Conference on Sensing, Communication, and Networking (SECON) (Stockholm: IEEE), 109–117. doi: 10.1109/SECON55815.2022.9918536

[B4] Beringer L. Petcher A. Katherine Q. Y. Appel A. W. (2015). Verified correctness and security of {OpenSSL}{HMAC},” in *24th USENIX Security Symposium (USENIX Security 15)* (Washington, DC), 207–221.

[B5] Chen C. Feng X. Zhou J. Yin J. Zheng X. (2023). Federated large language model: a position paper. arXiv [preprint]. arXiv:2307.08925. doi: 10.48550/arXiv.2307.08925

[B6] Chen G. Liu F. Meng Z. Liang S. (2022). Revisiting parameter-efficient tuning: are we really there yet? arXiv [preprint]. arXiv:2202.07962. doi: 10.48550/arXiv.2202.07962

[B7] Clark K. Khandelwal U. Levy O. Manning C. D. (2019). What does bert look at? An analysis of bert's attention. arXiv [preprint]. arXiv:1906.04341. doi: 10.48550/arXiv.1906.04341

[B8] Damgård I. Geisler M. Krøigaard M. Nielsen J. B. (2009). “Asynchronous multiparty computation: theory and implementation,” in International Workshop on Public Key Cryptography (Cham: Springer), 160–179. doi: 10.1007/978-3-642-00468-1_10

[B9] Damgård I. Jurik M. Nielsen J. B. (2010). A generalization of paillier's public-key system with applications to electronic voting. Int. J. Inf. Secur. 9, 371–385. doi: 10.1007/s10207-010-0119-9

[B10] Fowl L. Geiping J. Reich S. Wen Y. Czaja W. Goldblum M. . (2022). Decepticons: Corrupted transformers breach privacy in federated learning for language models. arXiv [preprint]. arXiv:2201.12675. doi: 10.48550/arXiv.2201.12675

[B11] Guo S. Wang Y. Li S. Saeed N. (2023). Semantic importance-aware communications using pre-trained language models. IEEE Commun. Lett. 27, 2328–2332. doi: 10.1109/LCOMM.2023.3293805

[B12] Houlsby N. Giurgiu A. Jastrzebski S. Morrone B. De Laroussilhe Q. Gesmundo A. . (2019). “Parameter-efficient transfer learning for NLP,” in International Conference on Machine Learning (Long Beach, CA: PMLR), 2790–2799.

[B13] Hu E. J. Shen Y. Wallis P. Allen-Zhu Z. Li Y. Wang S. . (2021). Lora: low-rank adaptation of large language models. arXiv [preprint]. arXiv:2106.09685. doi: 10.48550/arXiv.2106.09685

[B14] Hu S. Ding N. Zhao W. Lv X. Zhang Z. Liu Z. . (2023). Opendelta: a plug-and-play library for parameter-efficient adaptation of pre-trained models. arXiv [preprint]. arXiv:2307.03084. doi: 10.48550/arXiv.2307.03084

[B15] Huang W. Wang Y. Cheng A. Zhou A. Yu C. Wang L. . (2024). “A fast, performant, secure distributed training framework for LLM,” in ICASSP 2024-2024 IEEE International Conference on Acoustics, Speech and Signal Processing (ICASSP) (Seoul: IEEE), 4800–4804. doi: 10.1109/ICASSP48485.2024.10446717

[B16] Jiang F. Peng Y. Dong L. Wang K. Yang K. Pan C. . (2023). Large AI model empowered multimodal semantic communications. arXiv [preprint]. arXiv:2309.01249. doi: 10.48550/arXiv.2309.01249

[B17] Jiang F. Peng Y. Dong L. Wang K. Yang K. Pan C. . (2024). Large AI model-based semantic communications. IEEE Wirel. Commun. 31, 68–75. doi: 10.1109/MWC.001.2300346

[B18] Khowaja S. A. Nkenyereye L. Khowaja P. Dev K. Niyato D. (2024). Slip: self-supervised learning based model inversion and poisoning detection-based zero-trust systems for vehicular networks. IEEE Wirel. Commun. 31, 50–57. doi: 10.1109/MWC.001.2300377

[B19] Li B. Micciancio D. (2021). “On the security of homomorphic encryption on approximate numbers,” in Annual International Conference on the Theory and Applications of Cryptographic Techniques (Cham: Springer), 648–677. doi: 10.1007/978-3-030-77870-5_23

[B20] Li G. Wu J. Li S. Yang W. Li C. (2022). Multitentacle federated learning over software-defined industrial internet of things against adaptive poisoning attacks. IEEE Trans. Industr. Inform. 19, 1260–1269. doi: 10.1109/TII.2022.3173996

[B21] Li X. Wang S. Wu C. Zhou H. Wang J. (2023). Backdoor threats from compromised foundation models to federated learning. arXiv [preprint]. arXiv:2311.00144. doi: 10.48550/arXiv.2311.00144

[B22] Li X. Wu C. Wang J. (2024). “Unveiling backdoor risks brought by foundation models in heterogeneous federated learning,” in Pacific-Asia Conference on Knowledge Discovery and Data Mining (Cham: Springer), 168–181. doi: 10.1007/978-981-97-2259-4_13

[B23] Li X. L. Liang P. (2021). Prefix-tuning: optimizing continuous prompts for generation. arXiv [preprint]. arXiv:2101.00190. doi: 10.48550/arXiv.2101.00190

[B24] Liang C. Du H. Sun Y. Niyato D. Kang J. Zhao D. . (2024). Generative AI-driven semantic communication networks: architecture, technologies and applications. IEEE Trans. Cogn. Commun. Netw. 11, 27–47. doi: 10.1109/TCCN.2024.3435524

[B25] Lin B. Y. He C. Zeng Z. Wang H. Huang Y. Dupuy C. . (2021). Fednlp: benchmarking federated learning methods for natural language processing tasks. arXiv [preprint]. arXiv:2104.08815. doi: 10.48550/arXiv.2104.08815

[B26] Liu Y. Ott M. Goyal N. Du J. Joshi M. Chen D. . (2019). Roberta: a robustly optimized bert pretraining approach. arXiv [preprint]. arXiv:1907.11692. doi: 10.48550/arXiv.1907.11692

[B27] Lu X. Zhu K. Li J. Zhang Y. (2024). “Efficient knowledge base synchronization in semantic communication network: a federated distillation approach,” in 2024 IEEE Wireless Communications and Networking Conference (WCNC) (Dubai: IEEE), 1–6. doi: 10.1109/WCNC57260.2024.10571249

[B28] McMahan H. B. Moore E. Ramage D. Arcas B. A. (2016). Federated learning of deep networks using model averaging. arXiv [preprint]. arXiv:1602.05629. doi: 10.48550/arXiv.1602.05629

[B29] Paillier P. (1999). “Public-key cryptosystems based on composite degree residuosity classes,” in International Conference on the Theory and Applications of Cryptographic Techniques (Cham: Springer), 223–238. doi: 10.1007/3-540-48910-X_16

[B30] Pan S. Luo L. Wang Y. Chen C. Wang J. Wu X. . (2024). Unifying large language models and knowledge graphs: a roadmap. IEEE Trans. Knowl. Data Eng. 36, 3580–3599. doi: 10.1109/TKDE.2024.3352100

[B31] Peters M. E. Neumann M. Zettlemoyer L. Yih W.-t. (2018). Dissecting contextual word embeddings: architecture and representation. arXiv [preprint]. arXiv:1808.08949. doi: 10.48550/arXiv.1808.08949

[B32] Rivest R. L. Shamir A. Adleman L. (1978). A method for obtaining digital signatures and public-key cryptosystems. Commun. ACM 21, 120–126. doi: 10.1145/359340.359342

[B33] Samaniego M. Deters R. (2018). “Zero-trust hierarchical management in IOT,” in 2018 IEEE International Congress on Internet of Things (ICIOT) (San Francisco, CA: IEEE), 88–95. doi: 10.1109/ICIOT.2018.00019

[B34] Sun T. Shao Y. Qiu X. Guo Q. Hu Y. Huang X. . (2020). Colake: contextualized language and knowledge embedding. arXiv [preprint]. arXiv:2010.00309. doi: 10.48550/arXiv.2010.00309

[B35] Sun Y. Li Z. Li Y. Ding B. (2024). Improving lora in privacy-preserving federated learning. arXiv [preprint]. arXiv:2403.12313. doi: 10.48550/arXiv.2403.12313

[B36] Wang A. Singh A. Michael J. Hill F. Levy O. Bowman S. R. . (2018). Glue: a multi-task benchmark and analysis platform for natural language understanding. arXiv [preprint]. arXiv:1804.07461. doi: 10.48550/arXiv.1804.07461

[B37] Wang B. Li H. Guo Y. Wang J. (2023). Ppflhe: a privacy-preserving federated learning scheme with homomorphic encryption for healthcare data. Appl. Soft. Comput. 146:110677. doi: 10.1016/j.asoc.2023.110677

[B38] Wei K. Li J. Ding M. Ma C. Yang H. H. Farokhi F. . (2020). Federated learning with differential privacy: algorithms and performance analysis. IEEE Trans. Inf. Forensics Secur. 15, 3454–3469. doi: 10.1109/TIFS.2020.2988575

[B39] Wibawa F. Catak F. O. Kuzlu M. Sarp S. Cali U. (2022). “Homomorphic encryption and federated learning based privacy-preserving cnn training: COVID-19 detection use-case,” in Proceedings of the 2022 European Interdisciplinary Cybersecurity Conference (Ne wYork, NY: ACM), 85–90. doi: 10.1145/3528580.3532845

[B40] Wu C. Li X. Wang J. (2024). Vulnerabilities of foundation model integrated federated learning under adversarial threats. arXiv [preprint]. arXiv:2401.10375. doi: 10.48550/arXiv.2401.10375

[B41] Yang Z. Chen M. Li G. Yang Y. Zhang Z. (2024). Secure semantic communications: fundamentals and challenges. IEEE Netw. 38, 513–520. doi: 10.1109/MNET.2024.3411027

[B42] Zaken E. B. Ravfogel S. Goldberg Y. (2021). Bitfit: simple parameter-efficient fine-tuning for transformer-based masked language-models. arXiv [preprint]. arXiv:2106.10199. doi: 10.48550/arXiv.2106.10199

[B43] Zhang C. Li S. Xia J. Wang W. Yan F. Liu Y. . (2020). “{BatchCrypt}: efficient homomorphic encryption for {Cross-Silo} federated learning,” in 2020 USENIX annual technical conference (USENIX ATC 20) (Berkeley, CA), 493–506.

[B44] Zhang Z. Yang Y. Dai Y. Wang Q. Yu Y. Qu L. . (2023). “Fedpetuning: when federated learning meets the parameter-efficient tuning methods of pre-trained language models,” in Annual Meeting of the Association of Computational Linguistics 2023 (Toronto, ON: Association for Computational Linguistics), 9963–9977. doi: 10.18653/v1/2023.findings-acl.632

[B45] Zhao F. Sun Y. Feng L. Zhang L. Zhao D. (2024). Enhancing reasoning ability in semantic communication through generative ai-assisted knowledge construction. IEEE Commun. Lett. 28, 832–836. doi: 10.1109/LCOMM.2024.3365158

[B46] Zhou T. Yan H. Han B. Liu L. Zhang J. (2024). Learning a robust foundation model against clean-label data poisoning attacks at downstream tasks. Neural Netw. 169, 756–763. doi: 10.1016/j.neunet.2023.10.034 37981457

[B47] Zhu L. Liu Z. Han S. (2019). “Deep leakage from gradients,” in Advance in Neural Information Processing System, 32 (Vancouver, BC).

