# Correction: Synchronizing LLM-based semantic knowledge bases via secure federated fine-tuning in semantic communication

**DOI:** 10.3389/frai.2025.1758660

**Published:** 2025-12-16

**Authors:** Long Li, Yuanhang He, Rui Xu, Bei Chen, Boyu Han, Yuanyuan Zhao, Jianhua Li

**Affiliations:** 1 Shanghai Key Laboratory of Integrated Administration Technologies for Information Security, School of Computer Science, Shanghai Jiao Tong University, Shanghai, China; 2 National Key Laboratory of Security Communication, Chengdu, China

**Keywords:** semantic communication, large language model, semantic knowledge bases, homomorphic encryption, federated fine-tuning

The funder: National Key Laboratory of Security Communication Foundation [2023, 6142103042310] for Jianhua Li was erroneously omitted.

The correct **Funding** statement appears below.

The author(s) declared that financial support was received for this work and/or its publication. This work was funded by National Key Laboratory of Security Communication Foundation [2023, 6142103042310] for Jianhua Li.

The original version of this article has been updated.

